# Development and Challenges of Diclofenac-Based Novel Therapeutics: Targeting Cancer and Complex Diseases

**DOI:** 10.3390/cancers14184385

**Published:** 2022-09-09

**Authors:** Ayeman Amanullah, Arun Upadhyay, Rohan Dhiman, Sarika Singh, Amit Kumar, Dinesh Kumar Ahirwar, Ravi Kumar Gutti, Amit Mishra

**Affiliations:** 1 Cellular and Molecular Neurobiology Unit, Indian Institute of Technology Jodhpur, Jodhpur 342037, India; 2 Laboratory of Mycobacterial Immunology, Department of Life Science, National Institute of Technology, Rourkela 769008, India; 3 Division of Toxicology and Experimental Medicine, Department of Neuroscience and Ageing Biology, CSIR-Central Drug Research Institute, Lucknow 226031, India; 4 Department of Biosciences and Biomedical Engineering, Indian Institute of Technology Indore, Simrol, Indore 453552, India; 5 Tumor Microenvironment Unit, Department of Bioscience & Bioengineering, Indian Institute of Technology Jodhpur, Jodhpur 342037, India; 6 Department of Biochemistry, University of Hyderabad, Hyderabad 500046, India

**Keywords:** NSAIDs, diclofenac, COX, cancer, ageing, neurodegeneration

## Abstract

**Simple Summary:**

Diclofenac is a widely used drug for its anti-inflammatory and pain alleviating properties. This review summarizes the current understanding about the drug diclofenac. The potential applications of diclofenac beyond its well-known anti-inflammatory properties for other diseases such as cancer are discussed, along with existing limitations.

**Abstract:**

Diclofenac is a highly prescribed non-steroidal anti-inflammatory drug (NSAID) that relieves inflammation, pain, fever, and aches, used at different doses depending on clinical conditions. This drug inhibits cyclooxygenase-1 and cyclooxygenase-2 enzymes, which are responsible for the generation of prostaglandin synthesis. To improve current diclofenac-based therapies, we require new molecular systematic therapeutic approaches to reduce complex multifactorial effects. However, the critical challenge that appears with diclofenac and other drugs of the same class is their side effects, such as signs of stomach injuries, kidney problems, cardiovascular issues, hepatic issues, and diarrhea. In this article, we discuss why defining diclofenac-based mechanisms, pharmacological features, and its medicinal properties are needed to direct future drug development against neurodegeneration and imperfect ageing and to improve cancer therapy. In addition, we describe various advance molecular mechanisms and fundamental aspects linked with diclofenac which can strengthen and enable the better designing of new derivatives of diclofenac to overcome critical challenges and improve their applications.

## 1. Introduction

Non-steroidal anti-inflammatory drugs (NSAIDs) act by inhibiting eicosanoids resulting in anti-inflammatory, pain-relieving, and fever-reducing effects [1]. NSAIDs such as aspirin and indomethacin were synthesized before their therapeutic effects and mechanisms were clear. In 1971, John Vane suggested that the primary mechanism of the aspirin-like NSAIDs effect was cyclooxygenase (COX) enzymes inhibition [2]. Subsequently, improved understanding of the NSAID mechanism of action aided in the development of more NSAIDs, such as diclofenac, ibuprofen, and mefenamic acid. Different NSAIDs have distinct preferences for cyclooxygenase (COX) enzymes, some of which are non-selective to COX while others are specific COX-2 inhibitors [3]. COX enzymes, which are also called prostaglandin endoperoxide synthase, catalyzes the reaction of prostanoid formation (a subclass of eicosanoids), such as prostaglandins and thromboxane from arachidonic acid [4]. NSAIDs that interfere non-specifically with both cyclooxygenase-1 and cyclooxygenase-2 enzyme actions are grouped as non-selective COX inhibitors, whereas COX-2 specific NSAIDs are termed selective COX-2 inhibitors [5,6].

COX-2 inhibitors have an advantage over non-selective COX inhibitors due to a reduction in undesirable GI side effects, although they have been associated with an increased risk of thrombosis [7,8]. NSAIDs such as diclofenac, ibuprofen, and aspirin are common examples of non-specific COX inhibitors, whereas rofecoxib and celecoxib are categorized as selective inhibitors of cyclooxygenase-2 [5]. Progress in the knowledge of the NSAID mechanism of action has led to the identification of novel targets in addition to COX enzymes, associating them with their observed off-target effects [9,10]. However, few NSAID targets have also had potential therapeutic applications in diseases such as cancer and neurodegeneration [11,12,13,14].

Similar to other drugs, the discovery of diclofenac led to several studies elucidating its action mechanism, safety, off-target effects, and other pharmacological aspects [15,16]. Among NSAIDs, diclofenac is one of the most widely recommended [17]. Besides its use in conditions related to pain and inflammation, prior research have also found the role of diclofenac in the Wnt/β-catenin/T-cell factor pathway, Myc transport, and glucose metabolism, thereby suggesting its applications in cancer therapy [18,19]. The present review gives a comprehensive summary of the current understanding of the NSAID diclofenac. Additionally, findings that indicate the possibility of repurposing diclofenac therapy for diseases such as cancer and neurodegeneration are also discussed. Drug repurposing or repositioning is increasingly becoming an effective strategy in reinvestigating known old drugs for new therapeutic purposes, reducing the costs and time involved in de novo drug development [20,21,22]. Few recent examples include minoxidil, originally developed for hypertension, having effective outcomes in preventing hair loss [23]. Bromocriptine, developed for Parkinson’s disease, has been repositioned as a treatment for diabetes mellitus [24]. Similarly, thalidomide, initially developed to treat nausea in pregnant women, was granted approval as a multiple myeloma treatment with dexamethasone [25]. 

## 2. Diclofenac: Pharmacokinetics and Pharmacology

Diclofenac is a commonly recommended drug for pain, inflammation, and fever relief. The drug is used in conditions such as rheumatoid arthritis and pain related to surgery [26,27]. As the chemical name suggests, diclofenac is a phenyl acetic acid derivative available in the form of sodium, potassium, or sodium/misoprostol salt. Its molecular formula is C_14_H_10_C_l2_NNaO_2_, with a molecular weight of 318.14 [28]. It was first synthesized by Alfred Sallmann and Rudolf Pfister in 1973. In contrast to other classical NSAIDs, diclofenac is known to inhibit the COX-2 enzyme with greater efficiency than the COX-1 enzyme [29]. Diclofenac is a weak acid and has limited solubility in both aqueous and hydrophobic media [29,30]. Diclofenac is known to be absorbed completely and is directly proportional to the dose applied [31,32]. The peak plasma concentration is observed within a range of 10 min to 2 h, depending on the dosage form, viz., enteric coated tablets, solution, etc., and individual-based parameters such as gastrointestinal pH [33,34,35]. Diclofenac sodium salt is a gradually releasing formulation that has high dissolution in the high pH environment present in the duodenum in comparison with the low pH environment in the stomach [36]. The potassium salt of diclofenac was developed to increase the rate of diclofenac absorption, which could be used in conditions where rapid pain relief is needed [33]. Nearly sixty percent of intact diclofenac reaches the circulation [32,37]. 

Diclofenac metabolism extensively occurs in the liver, where conjugation of diclofenac to glucuronic acid takes place [38]. The conjugation to uronic acid is aided by the enzyme UDP glucuronosyltransferase-2b7 (UGT2B7) [39]. The resultant metabolite, acyl glucuronide, reacts with the sulfhydryl groups of proteins. Acyl glucuronide can be metabolized into 4-hydroxy diclofenac acyl glucuronide by enzyme cytochrome P4502C8 (CYP2C8), which forms benzoquinone imine, resulting in the oxidative bioactivation of diclofenac [40,41]. For the most part, this phenyl acetic acid drug metabolizes into 4′ hydroxyl metabolite, along with other minor metabolites, viz., 3′ hydroxyl metabolite and 5′ hydroxy metabolite [42]. Cytochrome P450 enzyme catalyzes the 4′ and 3′ hydroxylation, and cytochrome P450 3A4 catalyzes the formation of the 5′ hydroxyl metabolite [43,44,45]. More than 60% of the administered dose of diclofenac is excreted through the urine, whereas the remainder is removed as bile conjugates or metabolites of diclofenac [29]. The concentration of major metabolite, 4′ hydroxyl derivative, has been observed at levels around 30% in urine and 20% in bile [46]. As diclofenac has a short half-life of 2 h, repeated doses are required to maintain its plasma concentration to manage certain serious conditions [47].

**Figure 1:**
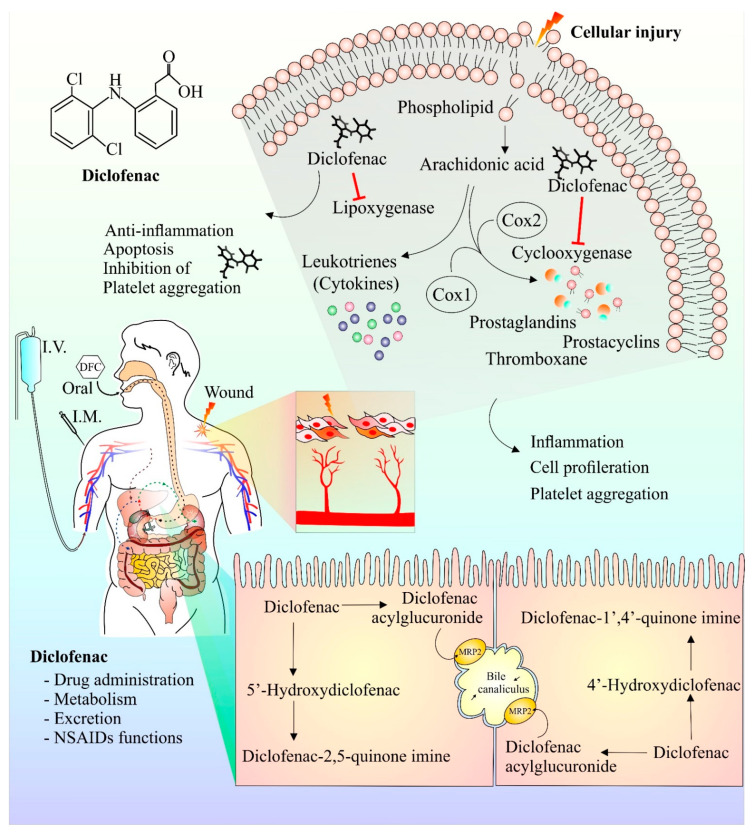
Chemical structure, mechanism of action, mode of drug administration, and metabolism of diclofenac. Cell damage results in release of arachidonic acid, a constituent of plasma membrane. Arachidonic acid in presence of enzyme cyclooxygenase and lipoxygenase is metabolized into prostaglandins, prostacyclins, and cytokines. These components are responsible for the generation of anti-inflammatory responses at the site of injury, causing pain and inflammation. Inhibition of platelet aggregation and cell proliferation by diclofenac have also been reported. The anti-inflammatory and analgesic effect of diclofenac is ascribed to its ability to hinder cyclooxygenase and lipoxygenase enzyme actions. Diclofenac metabolism mainly occurs in the liver and the major metabolic component of diclofenac is 4′ hydroxyl diclofenac metabolite. Other minor metabolites such as 5′ hydroxy diclofenac are also formed. The activation/formation and inhibition are depicted with arrows and blunt heads, respectively.

Following injury, membrane phospholipids are processed by phospholipase enzymes to form arachidonic acid [48]. Arachidonic acid is then further acted upon by COX and lipoxygenase enzymes to form prostaglandins and leukotrienes, respectively, that play key roles in the inflammatory and pain response [49]. Diclofenac has been observed to inhibit both the lipoxygenase- and COX-mediated inflammatory pathway [15]. Interestingly, diclofenac-mediated COX inhibition also has anti-proliferative characteristics [50]. In addition, diclofenac’s inhibitory effect on platelet aggregation has also been reported [51]. It has been observed that Mrp-2, a transport protein, plays a crucial part in the metabolism and transport of diclofenac. Rats deficient in Mrp-2 were much more resistant to the toxic intestinal effects of diclofenac [52,53]. Further studies investigating the transport and metabolism of diclofenac will help to design strategies that will improve efficacy and reduce toxicity. Figure 1 provides a schematic representation of diclofenac structure, currently available modes of its administration, its anti-inflammatory mechanism of action (upper right shaded region), and its hepatic metabolites (lower right shaded region). 

The majority of diclofenac is removed through hepatic transformation and nearly 1% is eliminated unchanged through the renal pathway [43]. Various pathways, such as inhibition of the thromboxane-prostanoid receptor, nitric oxide cGMP activation, antinociceptive mechanism, inhibition of PPAR gamma, regulation of ion channels, specifically potassium channels, and NMDA receptor inhibition were found to be affected by diclofenac, in addition to previously established cyclooxygenase and lipoxygenase pathways [15]. The analgesic effects of diclofenac on muscle pain is reported to be mediated by peripheral NMDA pain receptors [54]. The non-specific interactions of diclofenac on COX enzymes have been linked to its gastrointestinal side effects [55]. In addition to gastrointestinal (GI) complexities, various other undesired effects have been observed from off-target interactions of diclofenac. The pathways affected by diclofenac have been studied for the treatment of other diseases. Although these studies are still at a preclinical level, there is a potential for diclofenac to be used in other therapies. The side effects and therapeutic potential of diclofenac are discussed in the next section of the review.

## 3. Diclofenac: Side Effects, Adverse Drug Interactions, and Therapeutic Potential beyond Anti-Inflammation

### 3.1. Side Effects

The extensive understanding of the side effects of a particular drug is one of the most critical aspects of drug discovery. The undesired effects of a drug define the actual potential and limitations of a particular drug. As commonly known, NSAIDs that are non-specific inhibitors of COX enzymes, including diclofenac, are associated with the occurrence of GI side effects after long-term use. In the case of diclofenac, in addition to the GI complexities, other side effects observed include cardiological, hepatic, and renal toxicity [56,57,58]. Diclofenac has been found to exert prothrombotic effects by altering levels of PGI-2 and TxA 2 [59]. Additionally, diclofenac may also elevate the risks for heart attacks and myocardial infarctions [56,60]. A study comparing the cardiovascular risks of diclofenac with those of paracetamol, ibuprofen, and naproxen found that patients receiving diclofenac treatment had increased risk of cardiovascular events regardless of sex or age [61].

The use of topical diclofenac with DMSO on the knee of osteoarthritic patients has shown an occurrence of undesirable dermal reactions [62]. Some patients showed adverse GI effects, including diarrhea, nausea, abdominal pain, gastroesophageal reflux, and dyspepsia. Some patients also had undesired cardiovascular effects involving angina, hypertension, thrombosis, and myocardial infarction. Renal effects were also observed, as elevated creatinine levels were reported [62,63]. Further, in the case of a 35 year old patient, a severe life-threatening anaphylactic reaction to diclofenac was disclosed [64]. The side effects of diclofenac have been linked to its capacity to inhibit sodium and calcium channels in cardiac muscles [65] and change ROS levels [66,67]. Recently, diclofenac and other NSAIDs were found to interact with the Farnesoid X receptor that plays a crucial role in protecting the liver [10]. The toxic effects of diclofenac on the liver were also linked with lysosome dysfunction, mitochondrial injury, reactive oxygen species, modification in proteins due to drug metabolites, and immune-linked mechanisms [68,69]. Renal toxicity induced by diclofenac is attributed to mechanisms such as inhibition of renal prostaglandins [58] and destruction of proximal and distal tubules [70]. Recent findings have also shown that diclofenac can inhibit proteasomes, causing disturbance in proteostasis and mitochondrial dysfunction [71,72]. 

### 3.2. Adverse Drug Interactions

Adverse drug interactions have also been observed for diclofenac. Diclofenac, in combination with angiotensin-converting enzyme (ACE) inhibitors, have been found to cause increased systolic blood pressure, thereby reducing the effects of ACE inhibitors [73]. Cyclosporine A, used as an immunosuppressant in combination with diclofenac, has been found to cause increased risk of nephrotoxicity [74]. Aspirin reduces the uptake of diclofenac and a pharmacokinetic interaction exists between aspirin and diclofenac [75]. When used in combination with anti-coagulants, diclofenac has been found to increase bleeding complications [76]. The convulsive effects of quinolones, such as ciprofloxacin, has been observed to be elevated in the presence of diclofenac [77]. An incidence of renal failure followed by the death of a patient was documented when diclofenac was given with a dose of methotrexate [78]. Combinations of methotrexate and NSAIDs such as diclofenac, ketoprofen, and naproxen are reported to reduce excretion of methotrexate, leading to adverse drug–drug interactions [79]. Fixed-dose combinations of tramadol and diclofenac is not advised in patients suffering from serious renal impairment [80]. 

### 3.3. Therapeutic Potential beyond Anti-Inflammation

The off-target effects of diclofenac may also be utilized in the development of novel therapeutics. Diclofenac and meclofenamate sodium have been found to act as a novel voltage-gated potassium channel KCNQ2/KCNQ3 opener. The therapeutic application of this effect could be used for diseases associated with neuronal hyperexcitability, such as epilepsy [81,82]. In vivo work has shown encouraging results of diclofenac having an anticonvulsant effect. Diclofenac has been found to serve as a template for developing novel ion channel modulators [83]. Kv1.3 is also a target of diclofenac. This voltage-dependent potassium channel-mediated potassium-based current plays a key role in lymphocyte [84] and macrophage [85] activation. It has been observed that diclofenac reduces the immune response by affecting Kv1.3 channels [86], making diclofenac a good starting molecule for the development of autoimmune disorder therapeutics. The acid-sensing ion channel-1 is another diclofenac and NSAID target, which results in a reduction of the current induced by this acid-sensing channel [87]. This inhibition of current in sensory neurons has been thought to be another pathway of pain reduction, in addition to the classical prostaglandin inhibition by NSAIDs [87]. Diclofenac also inhibits phospholipase A2, which is considered to be the underlying mechanism through which diclofenac has therapeutic effects on acute pancreatitis [88]. However, indomethacin, another NSAID with a phospholipase A2 inhibitory effect, has been found to be the most potent agent in acute pancreatitis [89]. 

An interesting study reported that diclofenac could inhibit transthyretin amyloid fibril formation [90]. This property of diclofenac could be utilized in cases of senile systemic amyloidosis and familial amyloid polyneuropathy [91]. Inhibition of the AKR1C3 enzyme was found to be a therapeutic strategy in hormone-dependent cancers of the prostate and breast [92]. Diclofenac is one of the few known inhibitors of this enzyme. Further, it has been shown that diclofenac also has anti-bacterial and anti-mycotic effects. Diclofenac has been shown to inhibit the bacterial DNA replication of *Escherichia coli* and *Listeria monocytogenes* [93]. Altogether, in addition to the molecules involved in the COX-dependent anti-inflammatory pathway, the above studies provide a brief idea of other potential targets of diclofenac. These identified targets might have therapeutic value. Simultaneously, these targets must also be considered as causes of various side effects. Thus, a careful structural and metabolic study is required to overcome the existing limitations of diclofenac to properly utilize this drug in the development of therapeutics for other diseases.

Increased understanding of the pathways and mechanisms modified by diclofenac has provided crucial insights into diclofenac-mediated anti-nociceptive effects and its other possible applications. Diclofenac, like other NSAIDs, has been known to inhibit prostaglandin synthesis that results in its analgesic effects. Interestingly, diclofenac treatment is associated with the opening of potassium channels. The opening of potassium channels in afferent neurons is thought to produce anti-nociceptive effects [94]. Further, diclofenac-mediated inhibition of sodium currents in neurons has been found to play a crucial role in its anti-nociceptive outcomes [95]. Past studies have also shown evidence supporting diclofenac-mediated neuroprotective effects. The repressing effect of diclofenac on proton-induced currents in hippocampal interneurons may have a beneficial role in acidification-linked neuropathological conditions [96]. 

**Figure 2:**
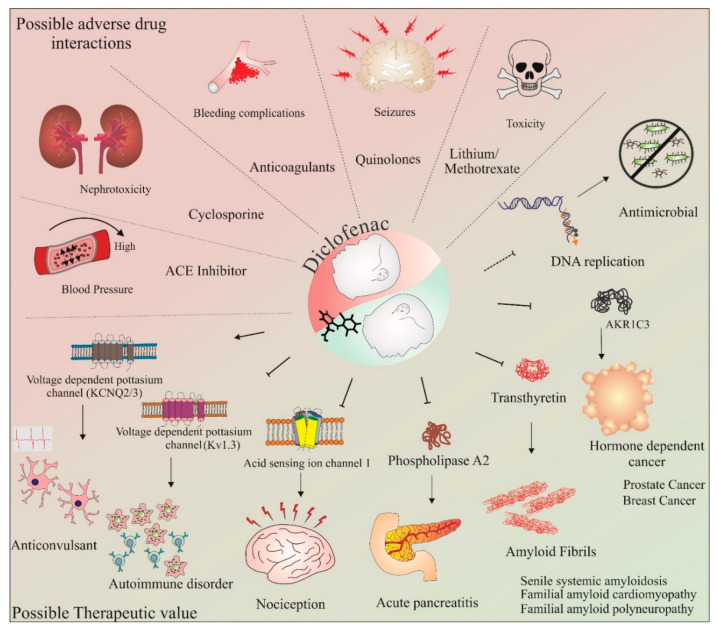
Therapeutic value and adverse drug–drug interactions of diclofenac. The potential use of diclofenac in combination therapies targeting various conditions has been assessed in numerous studies. However, some combinations of drugs with diclofenac have shown adverse effects. These include diclofenac in combination with ACE inhibitors, cyclosporine, anticoagulants, quinolones, and lithium/methotrexate. These combinations have resulted in increased blood pressure, nephrotoxicity, bleeding complications, seizures, and toxicity, respectively. In addition, with regard to its anti-inflammatory effects, studies have shown the therapeutic value of diclofenac in various diseases and conditions, such as autoimmune disorders, nociception, pancreatitis, amyloid fibril formation, seizures, cancer, and as an antimicrobial agent. The activation/formation and inhibition are depicted with arrows and blunt heads, respectively. The dotted blunt end represents an inhibitory effect that requires further investigations on diclofenac’s mechanism of action.

The potential of diclofenac as a neuroprotective agent and its involvement in the cell cycle and apoptosis is further discussed in the upcoming sections of this review. Some of the commonly known adverse drug–drug interactions and possible therapeutic applications of diclofenac are shown in Figure 2. These studies have provided important data with respect to the deleterious consequences of concurrent use of this drug. Thus, the adverse drug–drug interactions of diclofenac must be carefully assessed when designing combinatorial therapy with this drug. Further, as gender and individual differences may influence individual drug responses, methods such as utilizing individual donor characteristics possessing monocyte-derived hepatocyte-like cells may be beneficial in assessing these adverse drug–drug interactions on an individualistic basis [97]. Recently, using this method, enhanced toxicity while using diclofenac in combination with steroid hormones was observed in four out of nine patients [98]. However, aside from the adverse drug–drug interactions, the novel targets and applications identified so far (as depicted in Figure 2) make diclofenac an exciting molecule for repurposing this anti-inflammatory drug for other diseases. The following section provides a brief overview of our current understanding of the neuroprotective effects of diclofenac.

## 4. Neuroprotective Abilities of Diclofenac: A Unique Possibility

**Figure 3:**
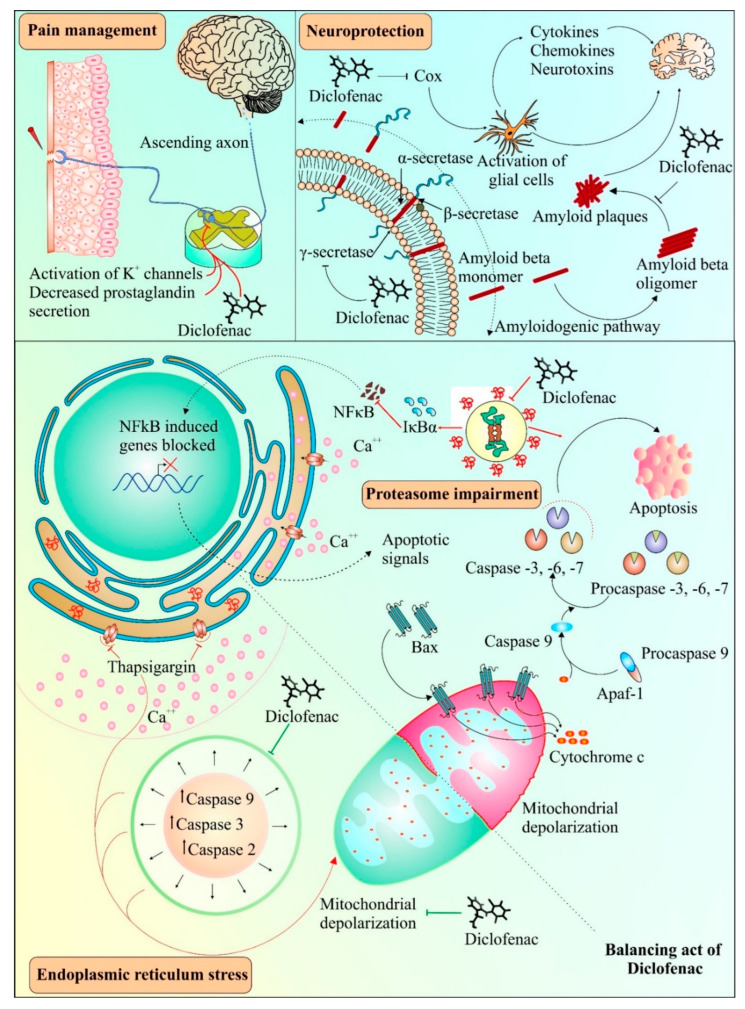
Possible and known applications of diclofenac. Diclofenac can act as an analgesic agent by activating transient outward potassium channels in neurons and simultaneously reducing the production of prostaglandins. Diclofenac can also act as a potent inhibitor of oligomerization of β-amyloid fibrils and plaque formation, which can be used in the development of therapies for diseases involving amyloid aggregation. Alternatively, diclofenac also interferes with the activation of glial cells, which may further contribute to its neuroprotective properties. Diclofenac has both inhibitory and inducing effects on cell death under different conditions. Endoplasmic reticulum stress generated by thapsigargin leads to activation of caspases and causes mitochondrial depolarization. Diclofenac suppresses the intrinsic pathway of apoptosis by interfering with caspase activation and mitochondrial depolarization. On the other hand, diclofenac treatment can result in proteasomal dysfunction generating downstream apoptotic signals such as mitochondrial cytochrome c release, causing stimulation of caspases leading to apoptosis. The pathway shown under dotted dual faced arrow curve in the top right shows the role of diclofenac in the inhibition of β-amyloid fibrils and plaque formation. The activation/formation and inhibition are depicted with arrows and blunt heads, respectively. The green color coding on arrows and blunt ends depicts the cell survival, whereas, the red color indicates cell death promoting signals.

Diclofenac has been found to have neuroprotective properties. A recent study suggested the neuroprotective role of diclofenac in chlorpromazine-induced catalepsy, having implications for Parkinson’s disease [99]. Previously, diclofenac had been shown to effectively impede Aβ 1-42 oligomerization and fibrillation, similar to another anti-inflammatory drug, celecoxib [100]. Misfolded transthyretin, a transport protein, can form aggregates in regions such as the heart and peripheral nerves and has been linked with amyloid diseases [101]. Diclofenac and its analogues have been found to inhibit aggregation of transthyretin amyloid [90]. Pancreatic amyloidosis includes islet amyloid peptide (IAPP) misfolding along with the occurrence of type 2 diabetes [102]. It was observed that diclofenac resulted in the inhibition of the oligomerization of the islet amyloid peptide (IAPP), causing a reduction in its cytotoxic effects [103]. Interestingly, the protective effect of diclofenac has been reported in protein misfolding diseases, such as Alzheimer’s [104]. Activation of glial cells by COX enzymes is known to be a common inflammatory mechanism in the brain, comprising cytokines, chemokines, and neurotoxins [105]. The impeding effect on COX enzymes by diclofenac is thought to aid in protecting brain cells from inflammation-induced toxicity [106]. Following an experimental focal penetrating traumatic brain injury in rats, the COX-2 inhibition-mediated protective effect of diclofenac appeared to be involved in reduced apoptosis and wound area [107]. Figure 3 provides a schema of various mechanisms involved in diclofenac-mediated protective effects.

## 5. Diclofenac: Several Targets, Multiple Outcomes

Prior studies have suggested both pro- and anti-apoptotic effects of diclofenac [72,108,109]. As depicted in Figure 3, diclofenac alleviates apoptosis induced by thapsigargin-induced endoplasmic reticulum stress conditions [110]. Moreover, the suppression of caspase activity by diclofenac was due to the inhibitory effect of diclofenac on mitochondrial depolarization, and subsequently, on apoptosis [110]. Interestingly, various pathways of diclofenac inducing apoptosis and inhibiting proliferation of cancer cells have also been reported, as shown in Figure 3, indicating its dual role. These mechanisms include inhibition of proteasomes [72], enhancement of ROS production [111], increase in p73 activity [112], and inhibition of MYC expression and lactate transport [19]. The next section discusses the effect of diclofenac on apoptosis and the cell cycle. Prior studies found that deregulated or impaired cell cycles and apoptosis played a crucial role in cancer and metastasis development [113,114,115]. Thus, apoptosis and cell cycle mechanisms have long been considered central mechanisms for novel therapeutic development against tumor development and metastasis [116,117]. Therefore, drugs affecting the cell cycle or apoptosis may have cytostatic or cytotoxic effects leading to the control of cancer cell proliferation and metastasis [118,119,120].

To date, different naturally isolated and laboratory-synthesized molecules have been developed as anticancer agents [121,122,123]. Some of the synthetic molecule structures have been derived from its natural analogs, such as topotecan from camptothecin [124], whereas some anticancer drugs, such as doxorubicin and epirubicin, were developed from a natural molecule, daunorubicin, commonly found in Streptomyces bacteria. An important limitation in designing new anticancer drugs is their off-target effects and limited knowledge of their pharmacokinetics profile. An important strategy to overcome such problem is drug repurposing. Drug repurposing involves the application of a known drug for other diseases [22]. Examples include ropinirole, a Parkinson drug repurposed for restless leg syndrome; gabapentin, an anti-epileptic drug repurposed for neuropathic pain; and methotrexate, an anticancer drug repurposed for arthritis [125,126,127]. Diclofenac has also been investigated for its therapeutic role in several other diseases. As mentioned in the previous section and shown in Figure 2, various beneficial applications of diclofenac have been identified, such as its anticonvulsant and antimicrobial effects. In addition, the potential of diclofenac as an anticancer agent has also been studied and is discussed in the following section.

## 6. Diclofenac as an Anticancer Agent: Role of Cell Cycle Regulation and Apoptosis

**Figure 4:**
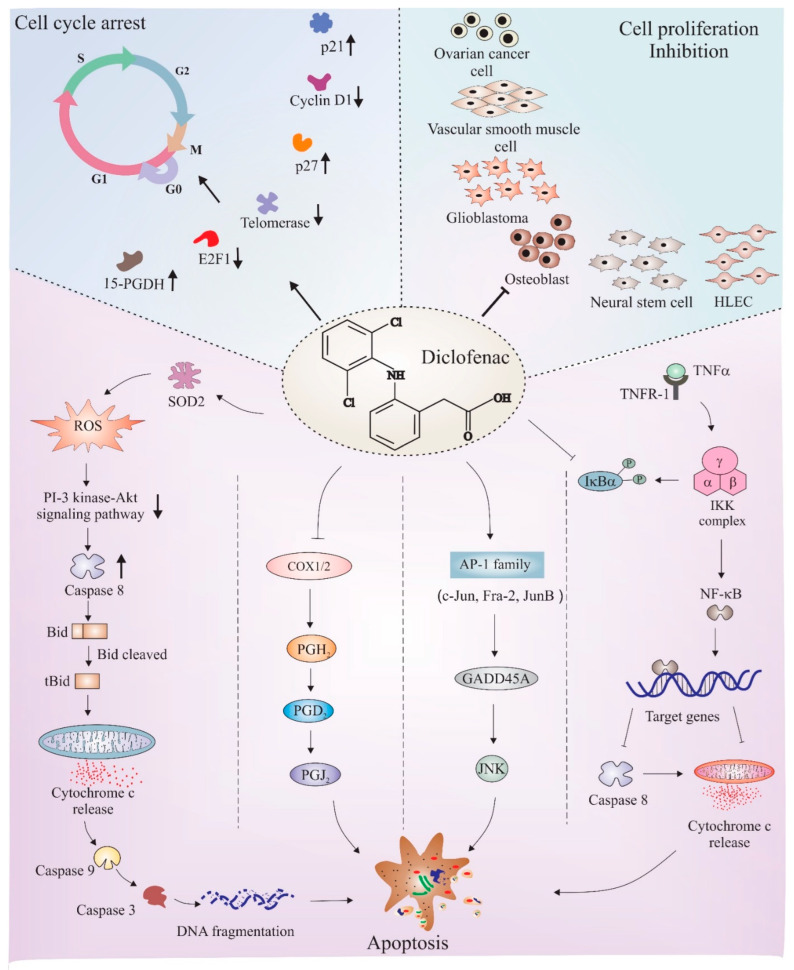
Schematic depiction of mechanisms of diclofenac-mediated apoptosis and cell cycle arrest. Diclofenac is found to obstruct cell cycle in different cell lines, such as neural stem cells, human lymphatic endothelial cells (HLEC), osteoblasts, glioblastomas (GBM), ovarian cells, and VSMC. Studies found an upregulation of cell cycle inhibitory proteins, such as p27, p21, and 15-hydroxyprostaglandin dehydrogenase (15-PGDH), and downregulation of inducers of cell proliferation, such as E2F1 transcription factor and cyclin D1, all crucial factors responsible for causing cell cycle arrest after diclofenac treatment. Diclofenac has also been shown to have pro-apoptotic effects. The major downstream mechanisms that have been thought to be the reason for diclofenac-mediated apoptosis includes ROS-induced downregulation of the PI 3-kinase/Akt signaling pathway, inhibition of NF-kB activity, disturbance in proteasome activity, interference with COX enzyme activity, and induction of the JNK pathway via AP1 transcription factors. The activation/formation and inhibition are depicted with arrows and blunt heads, respectively. The upward and downward small arrows in bold depicts elevation and depletion in corresponding proteins or pathway. The bold arrows or blunt ends (on top left and right sections) points to downstream proteins or cancer types, respectively indicating the effect of diclofenac. The normal arrows or blunt ends in lower section represents individual pathways known to be involved in diclofenac mediated apoptosis.

The anticancer properties of diclofenac have been studied in different cancers such as neuroblastoma [128], osteoblast [129], glioma [130], colorectal cancer [131], fibrosarcoma [132], and pancreatic cancer [133]. Being two major pathways, cell cycle and apoptosis mechanisms have been deeply studied to understand the manipulative role of diclofenac in these pathways. Figure 4 depicts a few major apoptotic and cell cycle proteins and pathways that are regulated by diclofenac. The following subsections discuss findings that link underlying cell cycle regulatory and apoptotic mechanisms associated with the anticancer effects of diclofenac.

### 6.1. Cell Cycle Regulation

Diclofenac treatment in glioma cells resulted in raised p21 expression, a cell cycle inhibitor, which is an effect associated with elevated levels of 15-PGDH (15 hydroxy prostaglandin dehydrogenase) [134]. Higher levels of p21 and p27 were also seen in vascular smooth muscle cells (VSMCs) treated with diclofenac. These outcomes were linked to the cell cycle inhibitory effect of diclofenac in the G1 phase of VSMCs [135]. Diclofenac also showed anti-carcinogenic effects in a colon cancer model. The anticarcinogenic effect of diclofenac was related with increased telomerase activity and depleted levels of CDK-4, CDK-2, cyclin E, and cyclin D1 [136]. E2f1, a transcription factor, is relevant for cancer progression and chemoresistance because it supports metastasis and invasion by inducing growth receptor signaling pathways when upregulated [137]. The anticarcinogenic outcome of diclofenac in ovarian cancer cells was suggested to be due to lowered expression of the E2f1 transcription factor [138]. Increased COX-2 levels were found to be the reason behind enhanced cellular proliferation and apoptosis resistance in various cancers [139,140]. Interestingly, diclofenac, a COX inhibitor, has been reported to have antitumor effects in rats with neuroblastoma [141]. 

### 6.2. Apoptosis

The influence of diclofenac on various apoptosis-inducing mechanisms has also been studied. The apoptotic effect of diclofenac in neuroblastoma cell lines was suggested to be linked with superoxide dismutase 2 (SOD2). SOD2 protects cell from ROS, and therefore, apoptosis or cellular damages [142,143]. Diclofenac impedes both expression levels and enzyme actions of SOD2 [128]. COX-2 has been found to inhibit the apoptosis mechanism activated through the Fas receptor; a receptor of the TNF family which induces apoptosis when it comes in contact with its agonists [144]. Further, a possible mechanism of the apoptotic effect of diclofenac is due to its COX inhibiting property, which causes inhibition of prostaglandins, such as prostaglandin H2 (PGH2), that have anti-apoptotic effects [15]. Another study by [145] proposed an activator protein-1-dependent mechanism of apoptosis induction by diclofenac in myeloid leukemia cells. The study proposed higher levels of activator protein-1 transcription factors, c-Jun, Jun-B, and Fra-2, which resulted in elevated levels of GADD45α, and therefore, JNK, leading to apoptosis. Another study found that the inhibition of TNF-α-mediated stimulation of NF-kB transcription factor activity by diclofenac sensitized hepatocytes towards apoptosis [146]. Taken together, various anticancer and pro-apoptotic mechanisms have been identified that supports the potential for diclofenac to be used as a therapeutic agent for different forms of cancer. 

## 7. Discussion

Diclofenac is one of the oldest and most widely utilized NSAID. Several studies have elucidated its pharmacokinetic and pharmacological properties. Various formulations of diclofenac have been developed, such as ointment, injections, tablets, etc., which can be utilized as needed. Interestingly, peripheral N-methyl D-aspartate (NMDA) receptor antagonism was also identified as a possible analgesic mechanism of diclofenac in a recent study [54]. Such findings suggest that diclofenac, in addition to its commonly known targets, i.e., COX enzymes, may have other useful targets as well. Some of these off-target effects have been related to the undesirable effects of diclofenac, such as gastrointestinal bleeding, cardiotoxicity, hepatotoxicity, etc. Recently, a new study suggested diclofenac had undesirable effects on the cornea through a p53-mediated apoptosis mechanism [147]. In addition, adverse drug–drug interactions of diclofenac, when tested in combination with other drugs such as ACE inhibitors, antimicrobial agents, anticoagulants, and cyclosporine, have also been reported. A recently concluded study in mice found the concomitant use of cefepime and diclofenac led to multiple organ failure [148]. The potential of diclofenac as an antimicrobial agent, anticonvulsant, amyloid inhibitor, and anticancer agent has been proposed and tested. However, the anticancer effects of diclofenac have not been extensively studied among these. 

Diclofenac shows anticancer properties in neuroblastoma, colorectal cancer, and fibrosarcoma [149]. However, most of these studies are at the preclinical level. A simple search on www.clinicaltrials.gov using diclofenac as a key term returns more than five hundred results. Out of these, more than fifty studies are under active status. These trials involve either only diclofenac or diclofenac in combination with other drugs as a therapeutic option for various conditions, such as common bile duct disease, diabetic oculopathy, various forms of cancers, etc. A further refinement of the search shows that around five studies are underway for the application of diclofenac in cancer. The outcome of trials such as NCT04091022 and NCT02636569, which are involved in understanding the effect of diclofenac on preventing or reducing the incidence of non-melanoma skin cancer may provide crucial information about the potential of diclofenac use in cancer therapy. A recent outcome from a clinical trial using topical diclofenac on actinic keratosis (AK) patients, a skin condition having altered keratinocyte proliferation, showed reprogramming of metabolism and immune cell infiltration in AK lesions [150]. Another ongoing clinical study involves diclofenac as a constituent of a four-drug combination, TL-118, i.e., cimetidine, metronomic cyclophosphamide, diclofenac, and sulfasalazine [149,151]. Thus, diclofenac may serve as an important constituent in cancer therapy when used with various other drugs. Recently, diclofenac as a curcumin adjuvant has been shown to aid anti-Alzheimer effects of curcumin in mice [152].

A limitation of diclofenac use is its associated side effects. Thus, to use or develop diclofenac in new therapies, studies are needed to develop novel strategies for reducing the side effects of diclofenac. It has been observed that a combination of proton pump inhibitors and diclofenac significantly decreases the risk of peptic ulcers [153]. Additionally, thymoquinone, a natural compound, reduces renal toxicity caused by diclofenac [154]. An interesting study that used vitamin B12 in combination with diclofenac showed that a reduced diclofenac concentration was needed to generate an analgesic effect [155]. The use of omega-3 fatty acids has also shown protective effects against diclofenac-induced hepatotoxicity [68]. The combination of diclofenac and the curcuminoid complex showed better tolerability and functional capacities in knee osteoarthritis [156]. Recently, a study proposed the development of diclofenac analogs with reduced hepatotoxicity [157].

The use of novel techniques in drug delivery can also aid in increasing the availability of the drug at a respective site, leading to reduced dose requirements. A study using polymeric nanosuspensions as a drug carrier showed improved ocular availability of diclofenac in rabbit eyes [158]. Similarly, another study used nanocrystal suspensions of diclofenac for skin inflammation and found that the anti-inflammatory effects were superior in comparison with existing commercial counterparts. The increased drug availability or amount at the inflammation site was thought to be a possible reason behind this [159]. A study using nanofibers obtained from the polymer of poly (D, L-lactide-co-glycolide) to deliver diclofenac locally, showed increased survival rates in a mouse model of oral carcinoma [160].Taken together, such studies may be useful in designing novel therapies that would effectively reduce the adverse effects of diclofenac.

## 8. Conclusions

In conclusion, the current literature shows many possibilities of diclofenac to be used therapeutically beyond its well-known role in pain management and anti-inflammation. Although there has been progress in gaining knowledge on the mechanisms of action and potential applications of this drug, major work is still at the preclinical level. To get a better understanding of the potential of this drug, more clinical studies are required. Being a well-known drug, repurposing diclofenac in diseases such as cancer or neurodegeneration could be of huge value and may expedite research being done to develop novel drug combinations for these complex diseases. 

## Abbreviations

AKR1C3	Aldo-keto-reductase family 1 member C3
Mrp-2	Multidrug resistance associated protein-2
NF-kB	Nuclear factor kappa light chain enhancer of activated B cells
NMDA	N-methyl D-aspartate receptor
PGI 2	Prostaglandin-I (2)
ROS	Reactive oxygen species
Tnf-α	Tumor necrosis factor alpha
TxA 2	Thromboxane-A (2)
